# Supplementary material for: Altered Envelope Structure and Nanomechanical Properties of a C-Terminal Protease A-Deficient Rhizobium leguminosarum
Source: Microorganisms. 2020 Sep 16;8(9):1421. doi: 10.3390/microorganisms8091421 (PMC7564917; doi:10.3390/microorganisms8091421)
Supplement: Supplementary file 1 [file microorganisms-08-01421-s001.pdf]

Supplementary Material for Publication

Altered envelope structure and nanomechanical properties of a C-terminal protease A-deficient *Rhizobium leguminosarum*

Dong Jun, Ubong Idem, and Tanya Elizabeth Susan Dahms

Table S1. HPLC retention times of major components from the muropeptide purification of the wild type 3841 and *ctpA* mutant 3845 strains. Peak numbers in bold were further analyzed by MALDI-TOF.

	Retention time (s)	
**Peak#**	**Wild type**	***ctpA* mutant**
1	15.8	15.9
**2**	16.7	16.8
**3**	17.5	17.6
4	18.4	18.5
5	20.0	20.0
**6**	20.9	20.9
7	24.8	ND
8	27.4	27.7
9	33.8	33.9
**10**	38.2	38.2
**11**	38.7	38.7
**12**	40.4	40.5
13	41.4	41.0
14	42.5	41.5
15	83.3	83.4
16	104.4	104.3
17	108.5	108.4
18	114.6	114.6
19	115.8	115.9
20	118.2	118.2
21	122.8	122.9

ND – no data

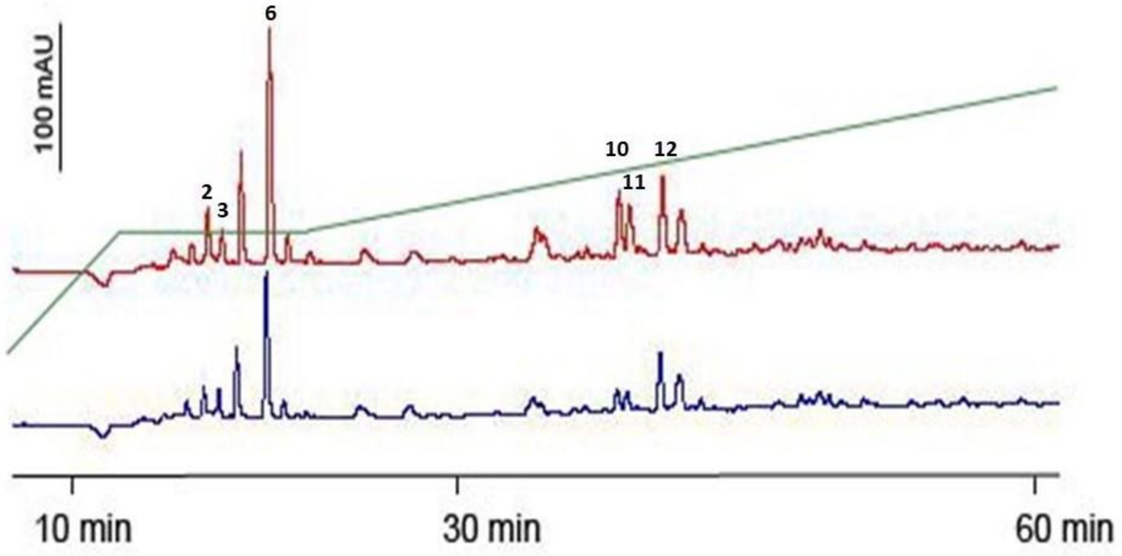

Figure S1. HPLC separation of muropeptides of wild type 3841 (Blue) and *ctpA* mutant 3845 (Red) as arbitrary absorbance units (mAU) over time (min). Optimized HPLC gradient of 1.7% to 7.8% acetonitrile with 0.1% TFA as the organic modifier is shown in green: 0% (0-5 min), 0-4% (5-12 min), 4% (12-22 min), 4-10% (22-82 min), 10-15% (82-91 min), and 15-50% (91-137 min) acetonitrile.

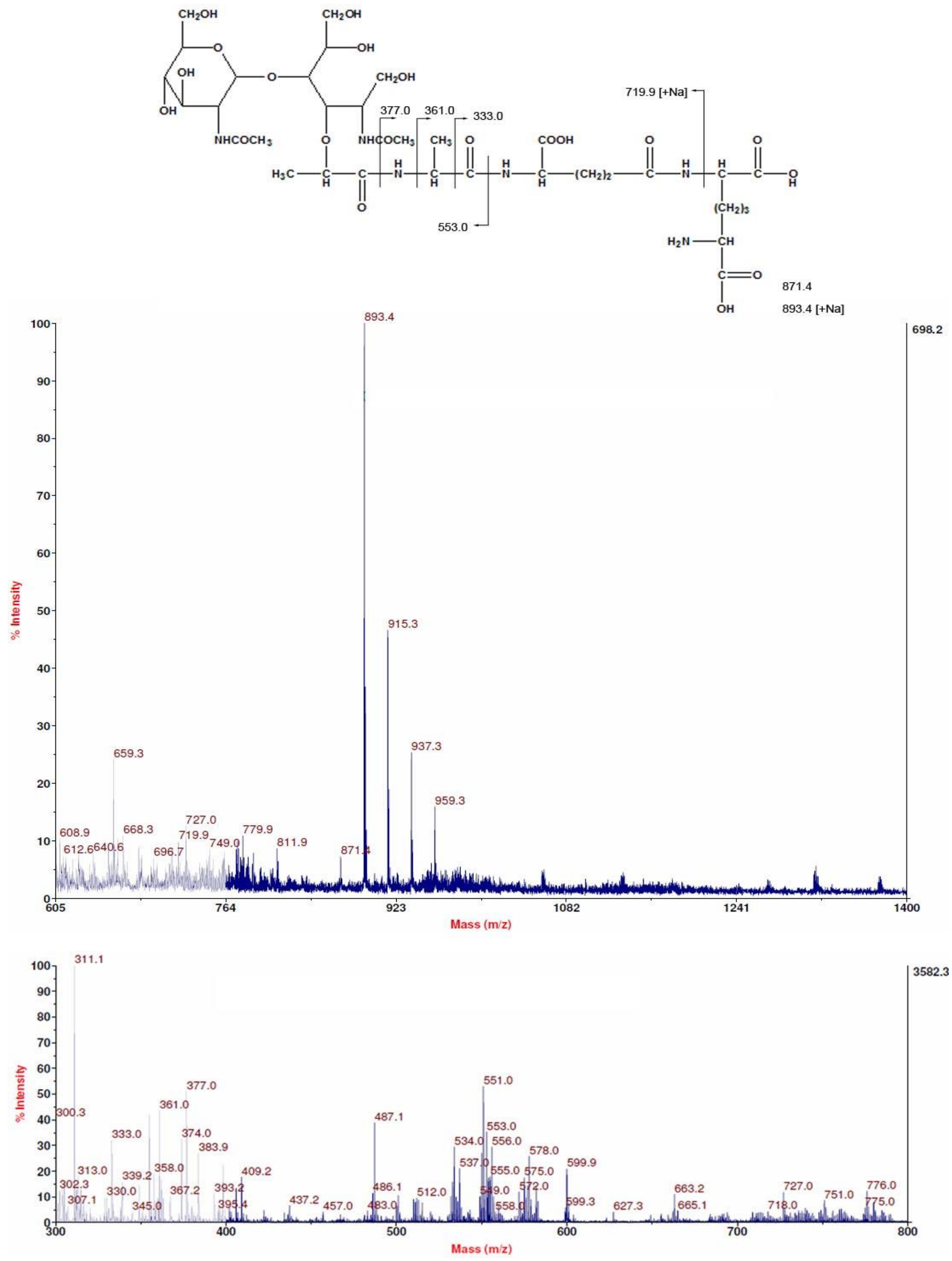

Figure S2. Representative MALDI-TOF mass spectra for 2 HPLC purified muropeptides from *Rhizobium leguminosarum* bv. *viciae* 3841.

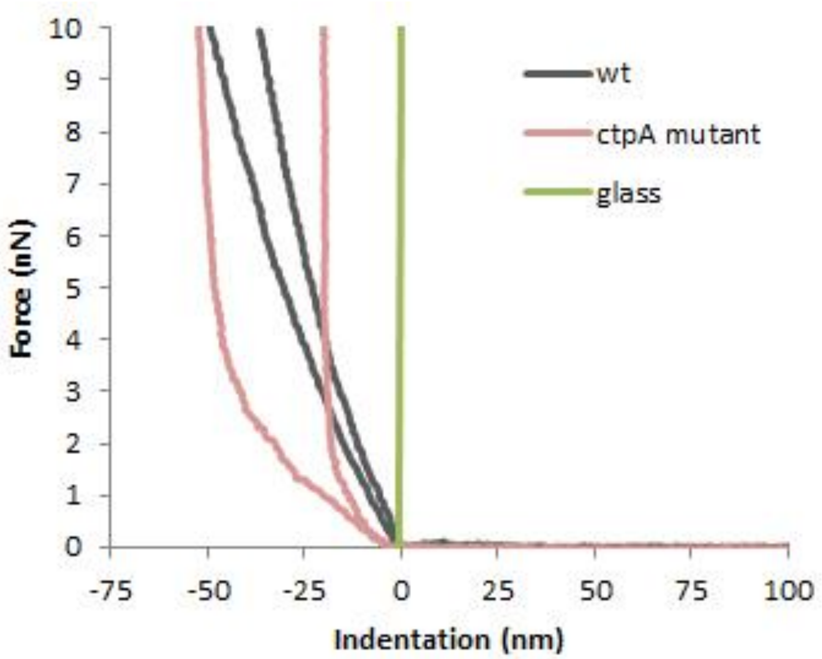

Figure S3. Representative approach indentation curves for *R. leguminsarum* bv. *viciae* 3841 (wt) and 3845 (*ctpA* mutant) on PLL-coated glass cover slips under water.

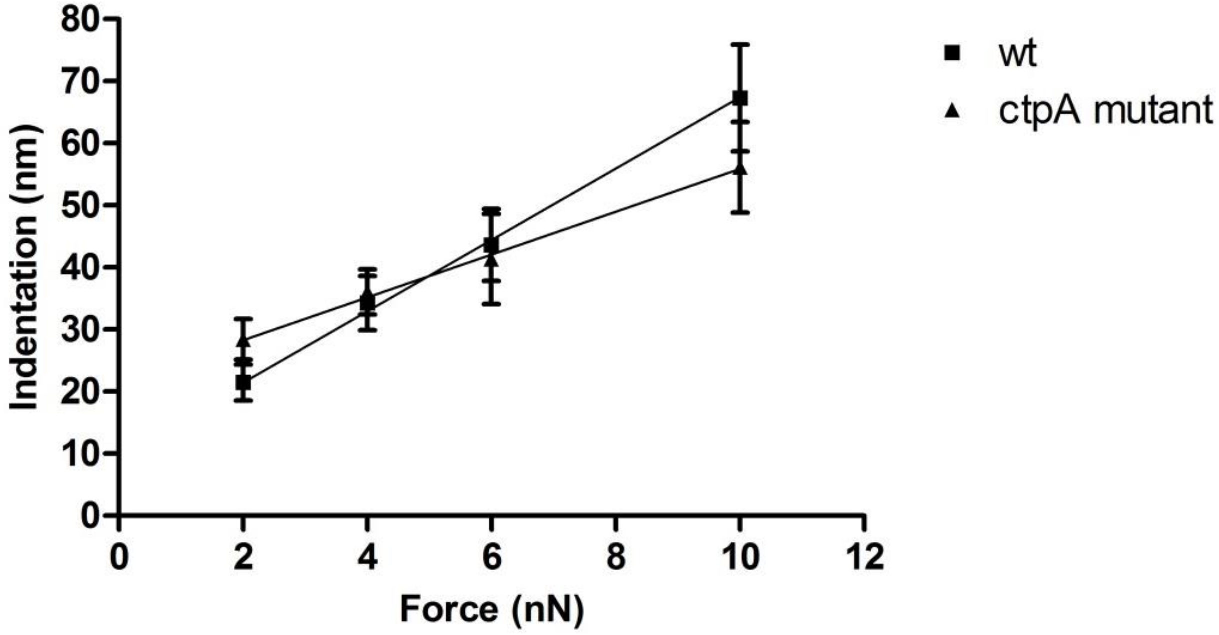

Figure S4. Cell indentation of *R. leguminsarum* bv. *viciae* 3841 (wt) (r = 0.998) and 3845 (*ctpA* mutant) (r = 0.997) on PLL-coated glass cover slips under water as a function of applied force.

A) B)

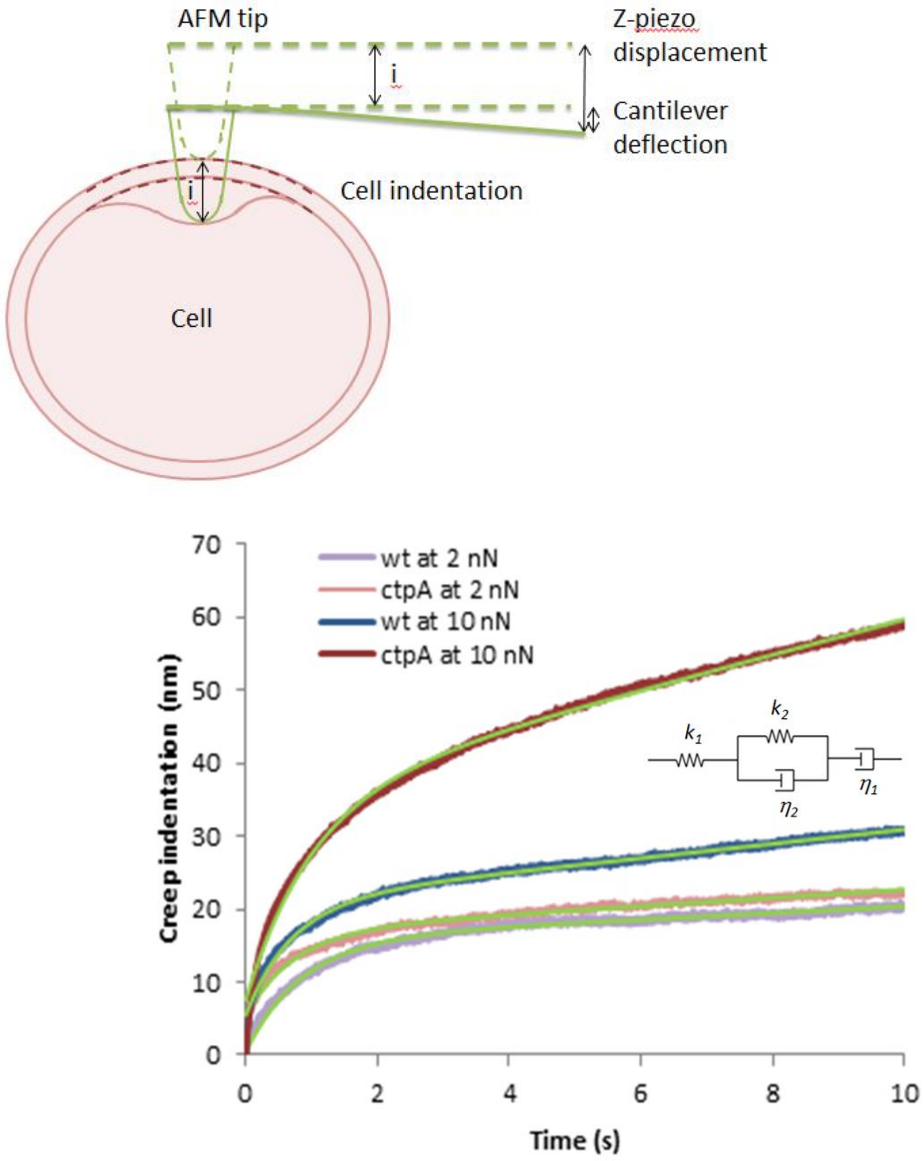

Figure S5. A) Schematic of a creep deformation experiment of a single cell by AFM, in which the AFM tip deforms the cell and gives rise to a Z-piezo displacement and cantilever deflection, used to calculate creep indentation (i). B) Creep deformation of *R. leguminsarum* bv. *viciae* 3841 (wt) and 3845 (*ctpA* mutant) at loading forces of 2 nN and 10 nN. The solid green line represents best fit to the data for a contact time of 10 s. Inset is a schematic diagram of Burgers' model in which k_1 and k_2 are spring constants and η_1 and η_2 are dashpot viscosities.

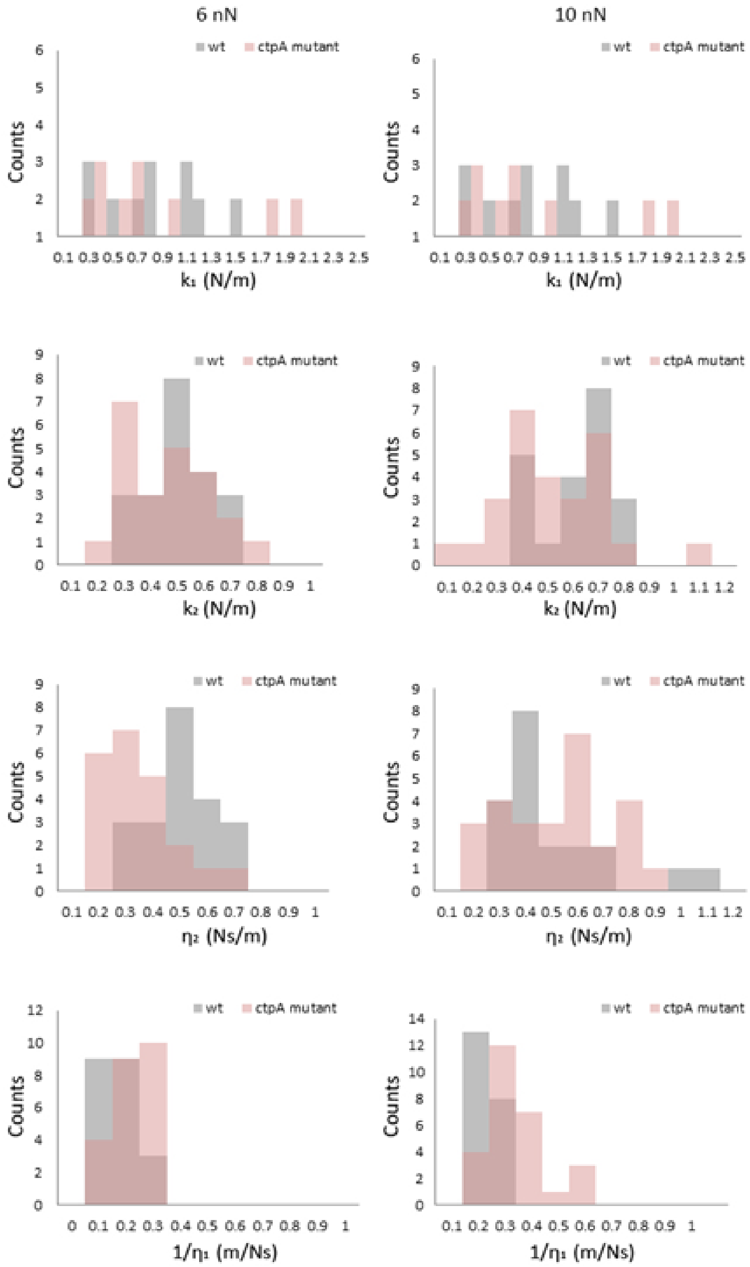

Figure S6. Histograms of viscoelastic parameters k_1, k_2, η_2, $1/\eta_1$ for *R. leguminsarum* bv. *viciae* 3841 (wt, grey) and 3845 (*ctpA* mutant, pink) at high (6, 10 nN) loading forces during creep deformation experiments. k_1 and k_2 are elastic spring constants, and η_1 and η_2 are the viscosity parameters from the Burgers' model, in which the Maxwell and Kelvin-Voigt models are connected in series.

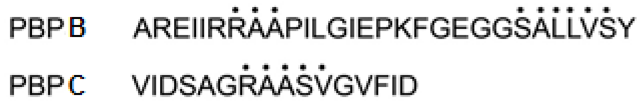

Figure S7. Potential C-terminal processing sites of PbpB and PbpC in *Rhizobium leguminosarum* bv. *viciae* 3841, based on substrate preference for Tsp (Prc, CtpA analog) from *E. coli*. The gene products of *pbpC* and *pbpB* in *R. leguminosarum* are homologous to PBP1C and PBP3 in *E. coli*, respectively.
